# Enhancing Therapeutic Efficacy of Donepezil, an Alzheimer’s Disease Drug, by *Diplazium esculentum* (Retz.) Sw. and Its Phytochemicals

**DOI:** 10.3390/ph17030341

**Published:** 2024-03-06

**Authors:** Woorawee Inthachat, Boonrat Chantong, Pornsiri Pitchakarn, Chawalit Takoon, Jirarat Karinchai, Uthaiwan Suttisansanee, Piya Temviriyanukul

**Affiliations:** 1 Food and Nutrition Academic and Research Cluster, Institute of Nutrition, Mahidol University, Salaya, Phuttamonthon, Nakhon Pathom 73170, Thailand; woorawee.int@mahidol.ac.th (W.I.); uthaiwan.sut@mahidol.ac.th (U.S.); 2 Department of Pre-Clinical and Applied Animal Science, Faculty of Veterinary Science, Mahidol University, Salaya, Phutthamonthon, Nakhon Pathom 73170, Thailand; boonrat.cha@mahidol.ac.th; 3 Department of Biochemistry, Faculty of Medicine, Chiang Mai University, Chiang Mai 50200, Thailand; pornsiri.p@cmu.ac.th (P.P.); jirarat.ka@cmu.ac.th (J.K.); 4 Mahidol University Frontier Research Facility (MU-FRF), Mahidol University, Nakhon Pathom 73170, Thailand; chawalit.tak@mahidol.ac.th

**Keywords:** Alzheimer’s disease, Ames test, *Diplazium esculentum*, donepezil, *Drosophila melanogaster*, human health, kaempferol, natural resource, quercetin, synergistic effect

## Abstract

Alzheimer’s disease (AD) is the most common type of dementia and a significant concern to global public health due to the prevalence of aging populations. Donepezil is one of only a few medications approved for use as an anti-AD agent but all have adverse side effects. Reducing the dosage of AD drugs with plant extracts (phytotherapy) while maintaining efficacy is one strategy to minimize adverse side effects. We previously reported the anti-AD properties of an edible fern, *Diplazium esculentum* (Retz.) Sw. (DE), which inhibited key enzymes involved in AD pathogenesis including acetylcholinesterase (AChE), butyrylcholinesterase (BChE), and β-secretase 1 (BACE-1). This study aimed to determine whether DE exhibited a synergistic effect with donepezil. The enzyme inhibitory assay showed that DE extract and its bioactive compounds, kaempferol, and quercetin, slightly impeded AChE inhibition with donepezil, while DE extract and quercetin showed synergistic or additive effects with donepezil against BChE and BACE-1, respectively. DE extract combined with donepezil also improved eye phenotypes in a *Drosophila* model of AD by preventing ommatidia atrophia and bristle breakages. Furthermore, the DE extract exhibited no genotoxic activities, as determined by the Ames test. Our data revealed that DE extract showed promise when combined with donepezil during AD treatment by targeting BChE and BACE-1.

## 1. Introduction

Alzheimer’s disease (AD) is the most common type of dementia. This neurological condition impairs thinking and memory skills, and causes loss of reasoning capacity and the inability to learn new skills and prioritize tasks that negatively impact day-to-day activities [1]. Data from Alzheimer’s Disease International estimated that 78 million people were living with AD worldwide in 2023 [2]. This complex disease induces harmful lifestyle factors; it is irreversible and requires extensive monitoring and treatment using a variety of drug types. Multiple hypothesized pathways have been documented to explain the pathogenesis of AD including: (i) overstimulation of the N-methyl-D-aspartate receptor (NMDA), a subtype of glutamate receptors, which leads to excessive stimulation of neurons [3]; (ii) the cholinergic hypothesis, which proposes rapid degradation of neurotransmitters (acetylcholine) responsible for the conduction of electrical impulses between neurons by acetylcholinesterase (AChE) and butyrylcholinesterase (BChE) [4]; and (iii) the amyloid hypothesis, which suggests that the cleavage of amyloid precursor protein (APP) by β-secretase 1 (BACE-1) results in the formation of neurotoxic, amyloid-β (Aβ) peptides [5]. Inhibition of NMDA receptor activation, AChE, BChE, BACE-1, and amyloid formation may be used together as a therapeutic agent for AD. The U.S. Food and Drug Administration (FDA) has approved some medicines to manage or treat AD symptoms. These medicines include donepezil, galantamine, memantine, rivastigmine, lecanemab, and aducanumab [6]. Donepezil, galantamine, and rivastigmine are cholinesterase inhibitors; memantine is an NMDA antagonist; while lecanemab and aducanumab are novel immunotherapies that reduce amyloid plaque. Investigations are currently underway into additional therapeutic approaches that hold promise, including nootropic drugs or medicinal plants [7,8], huperzine (a novel AChE inhibitor) [9], nerve growth factors (NGF) [10], and nuclear receptor peroxisome proliferator-activated receptor gamma (PPARγ) agonists [11]. Nevertheless, these therapies have not yet received approval.

Many adverse effects associated with AD treatments have been documented. Adverse effects include nausea, vomiting, diarrhea, exhaustion, muscle cramps, hepatotoxicity, disorientation, brain swelling, and bleeding [6,12]. Monotherapy (applying a single medication to treat an illness) is often successful at high dosages but has many undesirable side effects, while multiple action treatment mechanisms in combination with phytotherapy offer enhanced efficacy. Phytochemical treatment improves effectiveness through synergistic benefits, acceptability, and safety at lower dosages by particularly targeting disease progression [13,14]. As a result, using herbal medicines and phytochemicals to synergize with AD medications has recently been extensively researched since these approaches are safer, less expensive, and multi-target AD pathogenesis.

*Diplazium esculentum* (Retz.) Sw. (Pak-kood in Thai) is an edible fern belonging to the Athyriaceae family. The plant is commonly found in damp areas such as banks of rivers and canals, and is distributed throughout South Asia, East Asia, and Southeast Asia, including Thailand [15,16]. This fern is rich in minerals, nutrients, and phytochemicals including carotenoids, potassium, phosphorus, iron, phenolics, saponins, terpenoids, and flavonoids such as kaempferol and quercetin [17,18]. Some phytochemicals in *D. esculentum* have health-promoting properties and, historically, this plant has been used to alleviate a variety of human ailments through its anti-inflammatory, anti-diabetic, anti-microbial, and anti-AD properties [15,17]. Functional ingredients in *D. esculentum* hydro-alcoholic leaf extract exhibited a 50% lethal dose (LD_50_) in albino rats at ≥5000 mg/kg bw [19], with no changes in behavior, body weight, and blood biochemistry; this implies high extract safety.

We previously demonstrated that an ethanolic extract of *D. esculentum* (DE extract) strongly inhibited AChE, BChE, and BACE-1 activities in vitro. The extract also inhibited BACE-1 activities, decreased Aβ peptides, and improved locomotor function in a *Drosophila* model expressing human APP and BACE-1 representing the amyloid pathway, indicating promise for AD treatment.This study further elucidated the synergistic effects of *D. esculentum* and its identified phytochemicals, kaempferol, and quercetin with donepezil. All cholinesterase inhibitors have been reported to have the same efficacy in patients [12]. However, donepezil was chosen for this investigation because it inhibits AChE, BChE, and BACE-1 [20], rendering it an appropriate study target because DE extracts also inhibited these three enzymes. The synergistic properties of DE extract and its phytochemicals with donepezil against AChE, BChE, and BACE-1 were studied using the enzyme inhibition assay and were also tested by a robust in vivo assay employing *Drosophila melanogaster* expressing human APP and BACE-1 specifically in fly eyes. Findings revealed that the DE extract greatly enhanced the efficacy of donepezil by improving eye phenotypes. Information gained from this study sheds light on the advantages of DE extract and donepezil co-administration to reduce the concentration of donepezil and, thereby, also the side effects experienced by AD patients; however, further studies on animal models and clinical trials are required.

## 2. Results

### 2.1. Phenolic Profiles of Diplazium esculentum (DE) Ethanolic Extract Using Liquid Chromatography–Electrospray Ionization Tandem Mass Spectrometry

**Figure 1:**
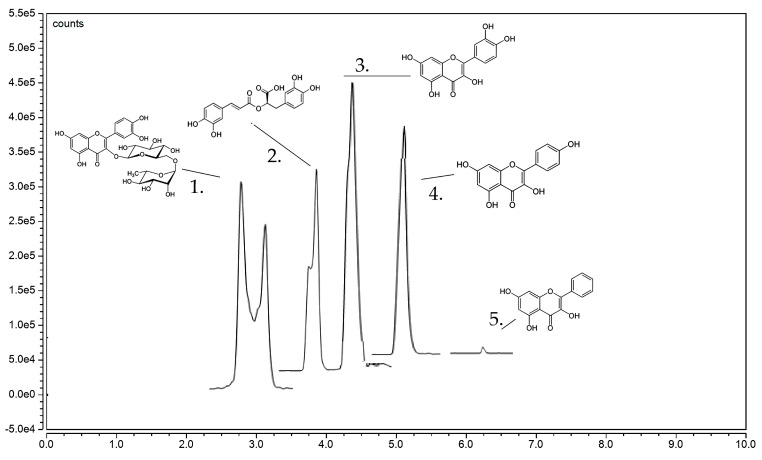
A liquid chromatography–electrospray ionization tandem mass spectrometry (LC–ESI–MS/MS) chromatogram of ethanolic extract of *Diplazium esculentum* (DE extract) presented five detected phenolic compounds including 1: rutin, 2: rosmarinic acid, 3: quercetin, 4: kaempferol, and 5: galangin. A full chromatogram with standard agents was shown in the Supplementary Figure S1.

Phenolic profiles of DE extract were investigated using a liquid chromatography–electrospray ionization mass spectrometry (LC-ESI-MS/MS), which is a hyphenated technique commonly employed in mass spectrometry analysis. This approach integrates the separation capabilities of high-performance liquid chromatography (HPLC) with enhanced mass accuracy provided by a mass spectrometer. Utilizing this technique, five compounds from thirty authentic standards including rutin, galangin, rosmarinic acid, quercetin, and kaempferol were identified in DE extract (Figure 1 and Table 1). Kaempferol was the most abundant at 167.67 mg/100 g dry weight (DW) followed by quercetin (1.9-fold lower), rosmarinic acid (3.1-fold lower), galangin (8.7-fold lower), and rutin (24.8-fold lower). Quercetin and kaempferol were chosen for the subsequent experiments.

### 2.2. Inhibitory Activities of the Key Enzymes Relevant to Alzheimer’s Disease

The half-maximal inhibitory concentration (IC_50_), which is defined as the concentration of the sample that inhibits 50% of enzyme activities, including two cholinesterase enzymes (AChE and BChE) and β-amyloid producing enzyme (BACE-1) were determined to study the synergistic effects between donepezil and DE extract, as well as its abundant bioactive compounds (kaempferol and quercetin). Various concentrations of donepezil, DE extract, kaempferol, and quercetin were subjected to AChE, BChE, and BACE-1 inhibitory assays. Results (Table 2) indicated that donepezil exhibited the lowest IC_50_ values followed by kaempferol, quercetin, and DE extract in all enzyme inhibitory assays. Donepezil exhibited the lowest IC_50_ value against AChE (1.30 µg/mL), which is lower than kaempferol (70.9-fold), quercetin (172.5-fold), and DE extract (2327.7-fold). Similar results were observed for BChE. Donepezil exhibited the lowest IC_50_ value against BChE (1.05 µg/mL); this was 50.5–2918.3-fold lower than the others. Meanwhile, the same trend in inhibitory activities was also detected in BACE-1. Donepezil exhibited an IC_50_ value of 0.55 µg/mL against BACE-1; this was 155.8–698.4-fold lower than the others. These results suggested that donepezil was a highly effective inhibitor against these enzymes, while the DE extract was the weakest.

### 2.3. Synergistic Effects between Diplazium esculentum (DE) Ethanolic Extract and Donepezil

**Figure 2:**
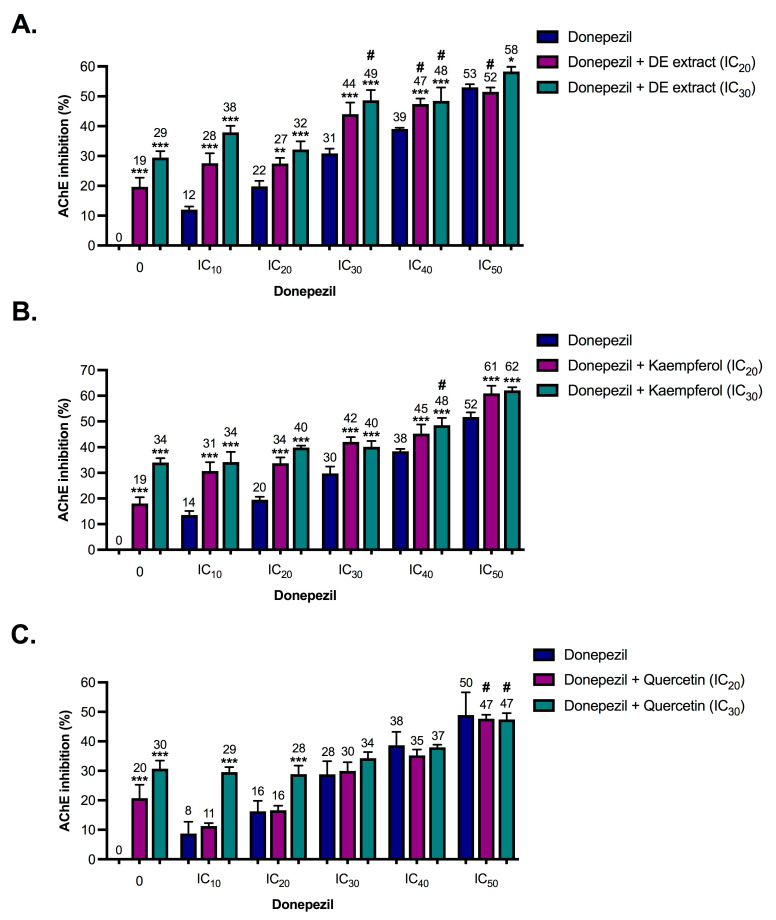
Acetylcholinesterase (AChE) inhibitory activities of donepezil (IC_10_ to IC_50_), ethanolic extract of *Diplazium esculentum* (DE extract) (IC_20_ and IC_30_), kaempferol (IC_20_ and IC_30_), quercetin (IC_20_ and IC_30_) and various combinations between (**A**) DE extract, (**B**) kaempferol and (**C**) quercetin. The values are mean ± SD of three independent experiments and statistical significance was analyzed against donepezil alone (dark blue) by one-way ANOVA followed by Tukey’s multiple comparisons test. * *p* < 0.05, ** *p* < 0.01 and *** *p* < 0.001. The combinations resulted in almost 50% AChE inhibition (indicated by the sharp symbol (#)) were selected for combination index (CI) calculation as shown in Table 3.

**Figure 3:**
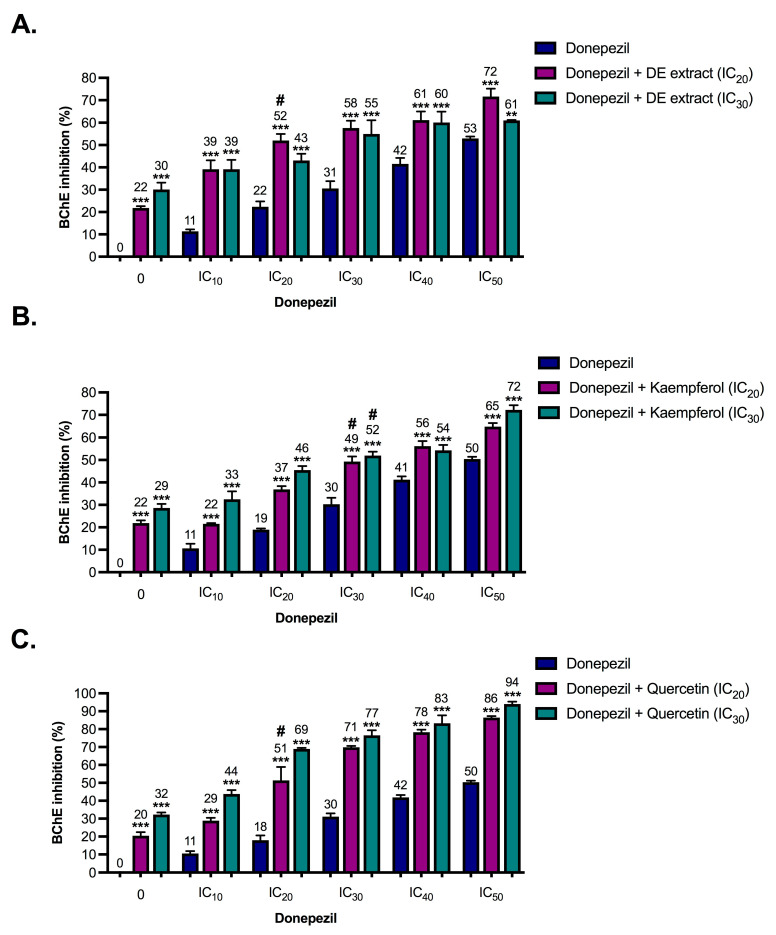
Butyrylcholinesterase (BChE) inhibitory activities of donepezil (IC_10_ to IC_50_), ethanolic extract of *Diplazium esculentum* (DE extract) (IC_20_ and IC_30_), kaempferol (IC_20_ and IC_30_), quercetin (IC_20_ and IC_30_) and various combinations between (**A**) DE extract, (**B**) kaempferol and (**C**) quercetin. The values are mean ± SD of three independent experiments and statistical significance was analyzed against donepezil alone (dark blue) by one-way ANOVA followed by Tukey’s multiple comparisons test. ** *p* < 0.01 and *** *p* < 0.001. The combinations resulted in almost 50% BChE inhibition (indicated by the sharp symbol (#)) were selected for combination index (CI) calculation as shown in Table 3.

**Figure 4:**
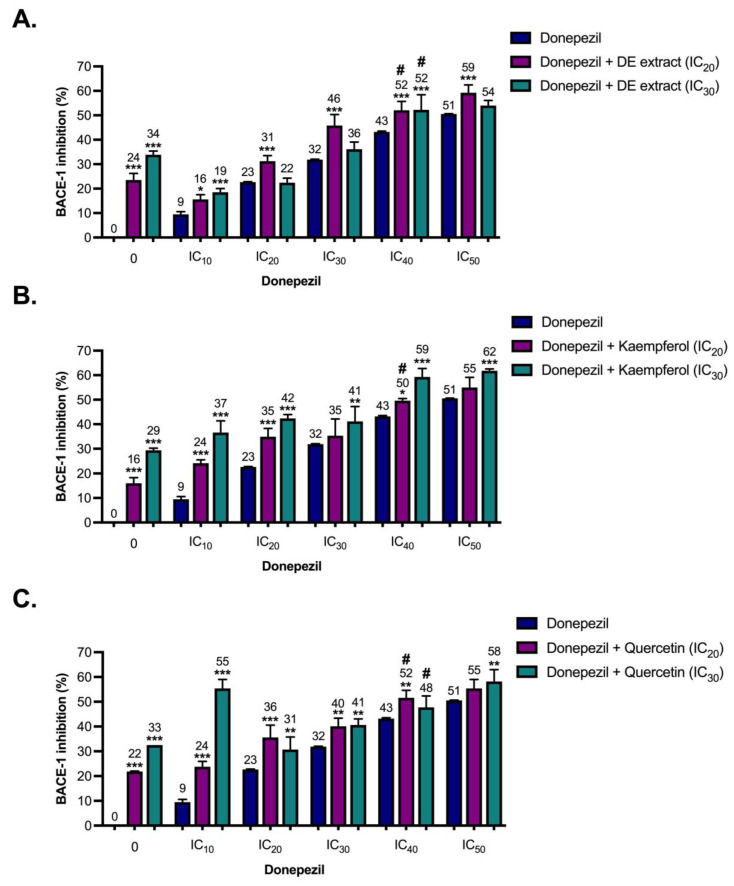
β-Secretase (BACE-1) inhibitory activities of donepezil (IC_10_ to IC_50_), ethanolic extract of *Diplazium esculentum* (DE extract) (IC_20_ and IC_30_), kaempferol (IC_20_ and IC_30_), quercetin (IC_20_ and IC_30_), and various combinations between (**A**) DE extract, (**B**) kaempferol and (**C**) quercetin. The values are mean ± SD of three independent experiments and statistical significance was analyzed against donepezil alone (dark blue) by one-way ANOVA followed by Tukey’s multiple comparisons test. * *p* < 0.05, ** *p* < 0.01, and *** *p* < 0.001. The combinations resulted in almost 50% BACE-1 inhibition (indicated by the sharp symbol (#)) were selected for combination index (CI) calculation as shown in Table 3.

The DE extract exhibited potential inhibition against the key enzymes in AD pathogenesis (Table 2). The synergistic effects between the DE extract phytochemicals (kaempferol and quercetin) and donepezil against AChE, BChE, and BACE-1 activities were then further evaluated. Two concentrations, including inhibitory concentration causing 20% and 30% enzyme inhibition, IC_20_ and IC_30_, respectively, of DE extract, kaempferol or quercetin were mixed with donepezil (inhibitory concentration causing 10% enzyme inhibition (IC_10_) to IC_50_) and subsequently assessed for inhibition of the enzymes AChE, BChE, and BACE-1. Figure 2, Figure 3 and Figure 4 show the enzyme inhibitory activities of donepezil (IC_10_ to IC_50_), DE extract (IC_20_ and IC_30_), kaempferol (IC_20_ and IC_30_), quercetin (IC_20_ and IC_30_), and various combinations against AChE, BChE, and BACE-1. Only combinations that resulted in almost 50% enzyme inhibition (indicated by the sharp symbols (#) in Figure 2, Figure 3 and Figure 4) were used to calculate combination index (CI) values.

As shown in Table 3, DE extract and its phytochemicals (kaempferol and quercetin) were slightly antagonized with donepezil to inhibit AChE. Addition of DE extract, kaempferol, and quercetin to donepezil resulted in very small changes in AChE inhibition (Figure 2), indicating the limited interference of DE extract, kaempferol, and quercetin in donepezil’s function. By contrast, DE extract, kaempferol, and quercetin demonstrated slight synergism with donepezil to inhibit BChE. Data showed that DE extract, kaempferol, and quercetin reduced donepezil concentration and inhibited BChE by almost 50% (Table 3). Donepezil also showed anti-BACE-1 activity [17,20]. The potential synergistic effects of donepezil in combination with DE extract, kaempferol, or quercetin were examined with respect to BACE-1 activities. Kaempferol and donepezil slightly antagonistically inhibited BACE-1, while DE extract and quercetin exhibited additive effects with donepezil (Table 3). The data indicated that decreased doses of donepezil when used concurrently with DE extract maintained the same level of enzyme inhibitory efficacy, particularly for BChE.

### 2.4. Effects of Diplazium esculentum (DE) Ethanolic Extract and Donepezil on Drosophila Eye Morphology

As previously noted, an ethanolic extract of *D. esculentum* (DE extract) improved AD phenotypes in a *Drosophila* model by suppressing BACE-1 and Aβ peptide, implying its potential as a synergistic agent with AD medicine [17]. Results in Table 3 showed that DE extract exhibited moderate synergism with donepezil against BChE and an additive effect with donepezil against BACE-1 in vitro. To further explore these findings in vivo, we selected flies expressing human APP and BACE-1 in the developing eyes as a screening model. These flies exhibited neurodegeneration in their retinas, rendering them suitable models for in vivo AD drug screening because their eye morphology was simple to examine and the test was rapid and reliable [21,22].

**Figure 5:**
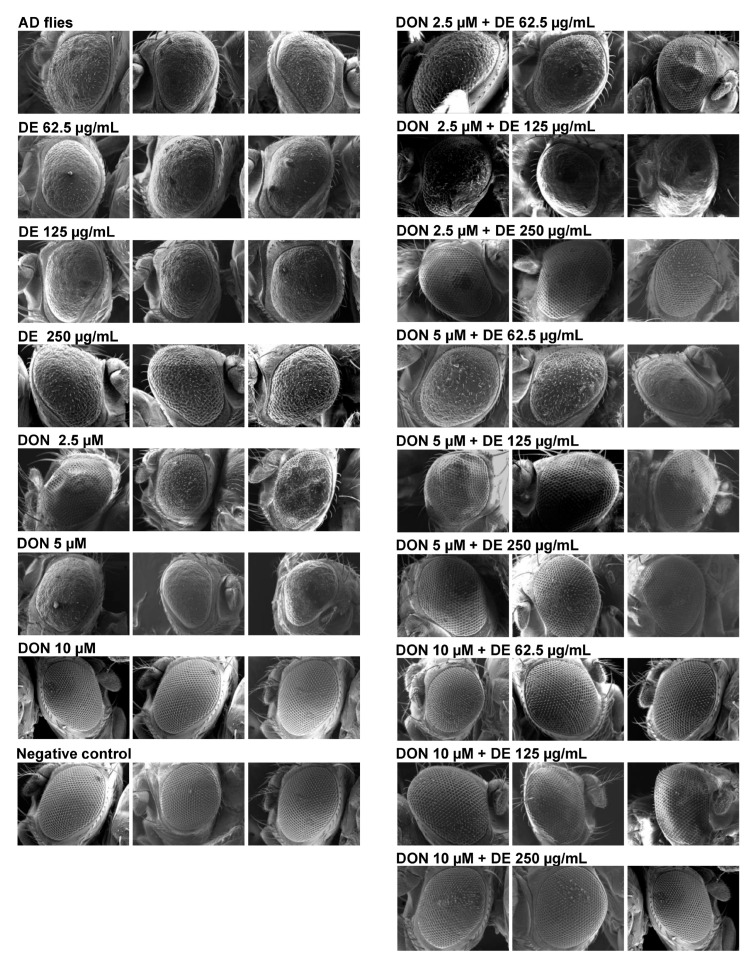
*Drosophila* eyes morphology captured using scanning electron microscopy (SEM). Alzheimer’s disease (AD) flies received various doses of donepezil (DON), ethanolic extract of *Diplazium esculentum* (DE) and their combinations for 21 days. AD flies refer to F1 progenies derived from crossing between GMR-GAL4 and UAS-APP-BACE-1, while negative control (AD-free flies) refer to GMR-GAL4 and without any treatment.

The flies were treated with different concentrations of DE extract (62.5, 125, and 250 µg/mL), donepezil (2.5, 5, and 10 µM), and various combinations of donepezil and DE extract, as illustrated in Figure 5. The concentrations of donepezil and DE extract utilized in this study were determined using our prior findings that demonstrated anti-AD effects in *Drosophila* models of AD at 250 µg/mL for DE extract and 10 µM for donepezil [17]. Figure 5 shows that the eyes of flies expressing APP and BACE-1 (AD flies) exhibited ommatidia atrophia and breakages of bristles in the whole eyes, while flies that did not express APP and BACE-1 (negative control, AD-free flies) showed intact ommatidia and bristles. This result suggested a strong neurodegeneration in the fly retina derived from APP and BACE-1. Donepezil (2.5 and 5 µM) and DE extract (62.5 and 125 µg/mL) at low concentrations did not reverse retina degeneration, while the structure of ommatidia and bristles was moderately enhanced by DE extract at a concentration of 250 µg/mL. However, this improvement was not as pronounced as when using donepezil at 10 µM, which exhibited eye phenotypes roughly similar to the negative control. The results implied the dose-dependent manner of DE extract and donepezil with improvement of eye phenotypes.

Results when combining the DE extract with donepezil are illustrated in Figure 5. A clear improvement in eye morphology was observed in three combinations including: (i) 2.5 µM donepezil + 250 µg/mL DE extract, (ii) 5 µM donepezil + 125 µg/mL DE extract, and (iii) 5 µM donepezil + 250 µg/mL DE extract. This suggests that the combination of DE extract with ineffective doses of donepezil (2.5 or 5 µM) yielded comparable outcomes to using 10 µM donepezil alone. An improvement in the eye morphology of AD flies was observed but this assay could not directly determine whether the effect was synergistic, additive, or antagonistic, unlike the enzyme assay. This preliminary investigation suggested that donepezil and DE extract inhibited eye degeneration via the neurotoxic pathway, amyloidogenesis, in vivo.

### 2.5. Genotoxicity Analysis of Diplazium esculentum (DE) Extract Using the Ames Test

As demonstrated, the DE extract showed potential for use as a synergistic agent with donepezil, despite being commonly ingested, with several safety factors requiring determination. Therefore, we preliminarily tested for the genotoxicity potential of DE extract using the Organization for Economic Cooperation and Development (OECD) guideline (OECD guideline for testing of chemicals No. 471 “Bacterial Reverse Mutation Test”) [23]. This guideline suggests that at least five *Salmonella typhimurium* strains should be used to cover several types of mutagens, such as point mutations and frameshift mutations. There are two types of mutagens: those that act directly and those that act indirectly, with the latter requiring bioactivation from the S9 extract to become mutagens. Results in Table 4 showed that all direct mutagens (4-NQO, NaN_3_, MMC, and 9-AA) had high revertant colonies compared to the negative control, with MR values ranging from 17.14 to 86.67. The mutagenic potential of chemicals is indicated when MR is ≥2. However, no increase in revertant colonies was observed in *Salmonella*-treated DE extract (10–2000 µg/plate) with MR at around 1 suggesting that the DE extract had no direct-acting mutagens. Experiments were also conducted involving incubation with the S9 extract (bioactivation). Results showed that 2-AA, a positive indirect mutagen, induced high revertant colonies in all the tested strains, with MR values ranging from 10.04 to 33.32. All five *Salmonella*-treated DE extracts showed no induction of revertant colonies when incubated with the S9 extract, as shown in Table 5. The findings suggested that the DE extract was devoid of mutagenicity and was genome safe.

## 3. Discussion

Alzheimer’s disease (AD) is an irreversible neurodegenerative disorder that induces dementia. AD drugs have been reported to develop side effects including nausea, diarrhea, exhaustion, hepatotoxicity, disorientation, and brain swelling [6,12]. Copious research has thus been conducted on the potential synergistic effects of herbal medicines combined with AD pharmaceuticals to reduce dosage quantity without compromising efficacy. Herbal medicines are of great interest because they are safe, easily assessable, and can act on several targets of AD pathogenesis. We previously reported that an ethanolic extract of *D. esculentum* (DE extract) significantly impacted AChE, BChE, and BACE-1 in vitro, suppressed BACE-1 activities and Aβ peptide formation, and improved locomotor functions of *Drosophila* expressing human APP and BACE-1 (an AD model of amyloidogenesis) [17]. These findings suggested the promising role of DE extracts as monotherapeutic agent against AD. Although the number of AD patients worldwide continues to rise, the development of new anti-AD medications is currently exceedingly difficult due to the failure of the majority of new AD drugs in large clinical trials [24]. In order to address this issue, adjuvant therapy or combination therapy involving plant extracts and approved AD medications may provide a solution [13,14]. Hence, this study further evaluated the efficacy of combining DE extract and its phytochemicals (kaempferol and quercetin) with the AD drug, donepezil, as a combination therapy both in vitro and in vivo. Results showed that DE extract and its phytochemicals, particularly quercetin, synergistically acted with donepezil to inhibit BChE and BACE-1, leading to reduced doses of donepezil for enzyme inhibition. While evidence from additional animal or clinical trials is required, the current study demonstrated the potential of DE extract as a combination therapy with donepezil, both in vitro and in vivo, for the first time. The DE extract was also tested for genotoxicity using the Ames test with data showing that the extract was genome safe.

Kaempferol and quercetin were the two main phytochemicals detected in the DE extract using LC–ESI–MS/MS, as shown in Table 1. Previous research reported on the phytochemical profiles of *D. esculentum*; in these the flavone glycosides, rutin and quercetin, and their aglycones are frequently mentioned [15,25]. However, in this study, kaempferol and rosmarinic acid were only detected in *D. esculentum* because phytochemical profiles can be affected by agro-climatic conditions, growth locations or even plant parts [26,27]. Ferns are rich in flavonoids and also contain various types of alkaloids such as lycodine, fawcettimine, diterpenoids, terpene glycosides, β-sitosterols, and bioflavonoids [28]. Further elucidation would assist in the identification of additional bioactive chemicals in *D. esculentum* with the potential to treat AD. Another challenge in the development of AD drugs is the requirement that bioactive compounds possessing neuroprotective properties penetrate the blood–brain barrier (BBB). Fortunately, kaempferol, quercetin, and rosmarinic acid—the top three phytochemicals found in DE (Table 1)—have also been reported to cross the BBB [29], highlighting the possibility that these compounds contributed to the rough eye improvement in *Drosophila* (Figure 5).

Different types of *D. esculentum* solvent extractions have been investigated regarding their pharmacological properties, especially for neuromodulatory effects both in vitro and in vivo [30]. *D. esculentum* ethanolic extract (1.25 mg/mL) inhibited AChE, BChE, and BACE-1 at 46.15, 53.12, and 55.91% [17], while a 70% methanolic extract inhibited AChE with an IC_50_ value of 272.97 µg/mL [31]. The in vivo experiments also indicated that ethanolic extracts of *D. esculentum* improved locomotor behaviors of Aβ-mediated toxicity *Drosophila* models by blocking BACE-1 activity and reducing Aβ42 peptide [17]. Utilizing an actophotometer, an aqueous leaf extract of *D. esculentum* stimulated the central nervous system (CNS) by increasing locomotor activity in albino mice treated with 100 mg/kg body weight [32]. These neuroprotective effects resulted from the plant’s bioactive compounds, quercetin and kaempferol. Kaempferol also inhibited AChE in a reversible mixed mode manner with an IC_50_ value of 12.43 µM [33], exhibited 2-fold higher inhibitory activity against BChE than AChE [34], and also delayed memory loss by maintaining climbing ability and reducing AChE activity in a transgenic *Drosophila* model for AD [35]. Quercetin also acted as a reversible mixed inhibitor against AChE with an IC_50_ value of 4.59 µM [36], while a 2.9-fold lower IC_50_ value against BChE was observed [37], suggesting greater quercetin inhibitory capacity against BChE. Quercetin also inhibited BACE-1 with an IC_50_ value of 0.55 µM [37], and demonstrated neuroprotective effects in Aβ_25–35_-induced oxidative stress in PC12 cells [36].

Results in this study showed that combinations of DE extract and its bioactive compounds (kaempferol and quercetin) as well as donepezil synergistically inhibited the activity of BChE and BACE-1 but had an antagonistic effect on AChE (Table 3). The beneficial effects of phytotherapy in animal models mimicking AD have also been reported. For example, an ethanolic fraction of *Melissa officinalis* leaf extract improved long-term memory in scopolamine-induced memory-impaired rats due to the strong transcription inhibition of AChE and BACE-1 genes in rat brains [38], while beneficial effects of combining phytotherapy in patients with AD were also reported. A combined therapy using *Ginkgo biloba* extract EGb 761 together with AChE inhibitors synergistically enhanced effectiveness and showed improved cognitive skills and neuropsychiatric symptoms in patients with moderate cognitive impairment (MCI) [39]. Combining phytotherapy with other drugs also enhanced treatment efficacy. Galantamine and citalopram, a selective serotonin reuptake inhibitor and an antidepressant, respectively, functioned synergistically to effectively inhibit BChE [40]. Thus, combining plant extracts or phytochemicals with AD drugs offers advantages to patients by producing synergistic benefits with lower dosages, while also offering additive effects that specifically target reducing disease development [13,14]. Regarding the cholinergic hypothesis, this study showed that DE extract, kaempferol, and quercetin acted antagonistically with donepezil against AChE, but synergistically with donepezil against BChE. As stated in the introduction, an acetylcholine neurotransmitter was degraded by both AChE and BChE. These two enzymes have comparable functions in AD pathogenesis but this does not render them equivalent as therapeutic targets for AD. AChE activities were reduced by 85–90% during AD progression, with the ratio between BChE and AChE in the cortical regions changing significantly from 0.2 to 1.0 [41,42], suggesting that AD patients had little residual AChE in the cortex [43]; meanwhile, BChE remained normal or even increased [40]. This result sheds light on the hypothesis that BChE may be the major enzyme contributing to acetylcholine reduction; thus, suppression of BChE has been considered as a promising treatment for AD [40,41]. However, a limited number of BChE inhibitors are available. Our results suggested that synergism between DE extract and its bioactive compounds (kaempferol and quercetin) with donepezil against BChE showed promise as an alternative therapeutic AD treatment by targeting BChE.

The potential for interaction between plant extracts and BACE-1 inhibitors is currently restricted due to the absence of an approved BACE-1 inhibitor. BACE-1 is a rate-limiting enzyme in neurotoxic Aβ peptide and senile plaque formation, a hallmark of AD [5]. BACE-1 has therefore been a desirable clinical target for AD treatment. This study showed that DE extract and quercetin (but not kaempferol) acted synergistically with donepezil against BACE-1. Notably, the data suggested that DE extract may have the potential to treat multiple targets of AD (cholinergic hypothesis via BChE and amyloid peptide pathway via BACE-1). Recently, increasing focus has concentrated on multi-target therapies as prospective alternative treatments for AD, with the recognition that AD—which has multifactorial etiology—may not be sufficiently addressed using the conventional single-target approach, also referred to as the “one compound-one target” approach [44]. Although we determined the antagonistic effect of kaempferol with donepezil against BACE-1, the DE extract improved rough eye phenotypes in flies expressing APP and BACE-1 (Figure 5), implying that either other bioactive substances may exhibit synergy with donepezil and contribute to the overall synergistic effect, or the antagonistic impact of kaempferol was relatively insignificant in the DE extract.

The safety aspect must also be considered to encourage the use of plant extracts. As previously stated, DE extract showed LD_50_ in rats at ≥5000 mg/kg bw; with no changes in behavior, body weight, and blood biochemistry indicating the safety of the extract [19]. However, genotoxicity testing for DE extract was limited. Thus, the DE extract was subjected to genotoxicity testing using the Ames test. Results showed that the DE extract did not induce DNA mutations in the five bacterial strains (Table 4 and Table 5), demonstrating its safety for further animal or clinical trials. Kaempferol and quercetin were the two principal agents in the DE extract and their mutagenic properties have been presented in several articles. Kaempferol demonstrated genotoxicity exclusively when the S9 extract was present in *S. typhimurium* TA98 and TA100; while, conversely, quercetin exhibited genotoxicity in the bacterial strains both with and without the S9 extract [45]. Kaempferol also induced nuclear DNA damages and lipid peroxidation in isolated rat liver nuclei [46], while quercetin induced micronuclei—a marker for DNA breaks—in the presence and absence of the S9 extract in V79 Chinese hamster lung cells and human lymphocytes [47]. Kaempferol was bio-transformed by Phase I enzyme into quercetin [48], providing insight into the reason why kaempferol requires the S9 extract to act as a mutagen. Intriguingly, kaempferol or quercetin-rich nutraceuticals such as kaempferol glycoside-rich roasted goji berry leaf extract, kaempferol aglycone-rich horseradish leaf extract, and dihydroquercetin-rich extract were not found to be mutagenic when investigated in animal models (in vivo) [49,50,51]. This result was confirmed by data from Takanashi et al. who showed that administration of kaempferol or quercetin in rats for 540 days did not significantly induce tumors compared with the control [52]. These findings suggested that (i) limited bioavailability in animal models reduced the mutagenicity potential of kaempferol and quercetin, and (ii) a combination of kaempferol or quercetin with additional phytochemicals may impede the mutagenic characteristics exhibited by these substances. These results serve as a basis for utilizing crude DE extract with kaempferol or quercetin standardization rather than a single ingredient to synergistically interact with donepezil.

## 4. Materials and Methods

### 4.1. Sample Preparation and Extraction

**Figure 6:**
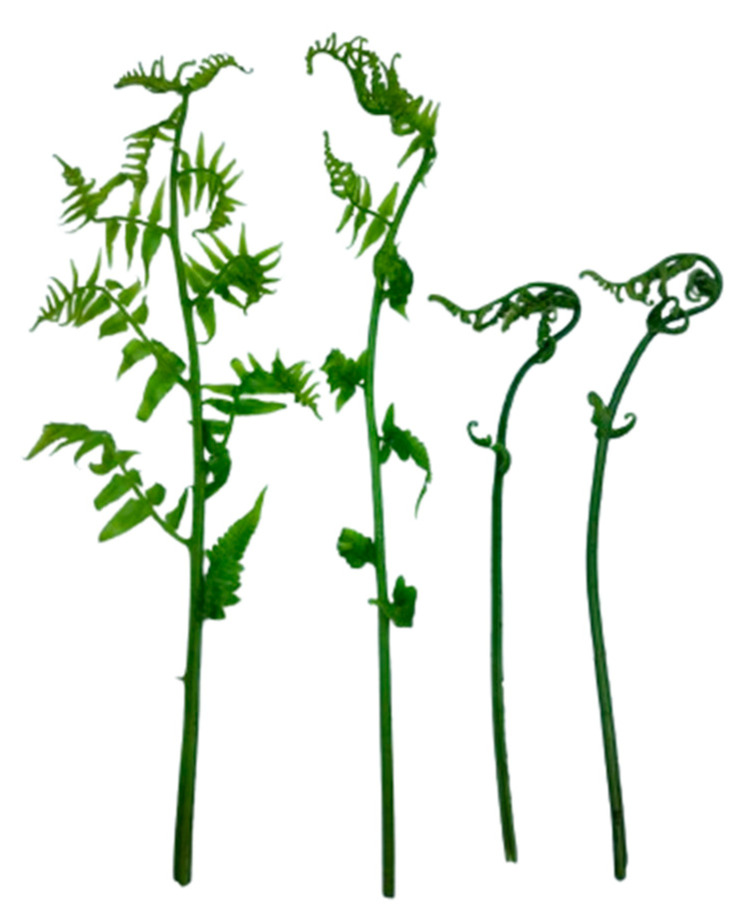
Photo representing *D. esculentum* used in the present study.

*Diplazium esculentum* (Retz.) Sw. (DE) (Figure 6) was collected from Chiang Mai, Thailand. The samples were identified by Dr. Kanchana Pruesapan (Taxonomist) and deposited at the Bangkok Herbarium (BK), Bangkok, Thailand (voucher specimens: BK069943). Young fronds were cleaned and cut into pieces. The samples were then freeze-dried using a Heto PowerDry PL9000 (Allerød, Denmark), and blended to a fine powder using a grinder (Philips 600 W series, Philips Electronics Co., Ltd., Jakarta, Indonesia). For extraction, one gram of DE per 10 mL of 80% ethanol was mixed and extracted at 37 °C for 6 h. The samples were then centrifuged at 3000× *g* for 20 min using a refrigerated centrifuge (Hettich^®^ ROTINA 38R, Andreas Hettich GmbH, Tuttlingen, Germany) and the supernatant was collected. An Eyela N-1200 Series rotary evaporator was used to remove the solvent. The dried extract was redissolved in DMSO, and stored at −20 °C until further analysis.

### 4.2. Phytochemical Analysis Utilizing Liquid Chromatography–Electrospray Ionization Tandem Mass Spectrometry (LC–ESI–MS/MS)

The LC–ESI–MS/MS was conducted according to our previous procedure without modification [53]. Briefly, 0.5 g of DE extract was mixed with 40 mL of formic acid and 10 mL of 62.5% (*v*/*v*) methanol containing 0.5 g tert-butylhydroquinone. Then, it was shaken for two hours in an 80 °C water bath shaker (TW20 series, Julabo GmbH, Seelbach, Germany). After incubation, we added 100 µL of ascorbic acid (1% *v*/*v*) to stop the reaction and filtered it through a 0.22 µM polytetrafluoroethylene (PTFE) membrane syringe filter. The LC-ESI-MS/MS system was used to identify phenolics and isoflavones profiles in the DE extract. The system included a Chromeleon 7 chromatography data system (version 7.2.9.11323) from Thermo Fisher Scientific, a Dionex Ultimate 3000 series ultrahigh-performance liquid chromatographer (UHPLC), a TSQ Quantis Triple Quadrupole mass spectrometer (MS), and a diode array detector (Bremen, Germany). The sample was loaded onto the LC-ESI-MS/MS system using a flow rate of 0.5 µL/min with 10 min run time for phenolics and 20 min run time for isoflavones. Using a 2.6 µm Accucore RP-MS column, 2.1 × 100 mm (Thermo Fisher Scientific, Bremen, Germany) and gradient mobile phase consisting solvent A and solvent B were acetonitrile and Milli-Q water respectively, both containing 0.1% formic acid (*v*/*v*). Gradient mobile phase: 0.0–8.0 min gradient of 90% B and 10% A; 8.0–8.1 min gradient of 20% B to 80% A; 8.1–10.0 min gradient of 90% B and 10% A for phenolic analysis [53,54].

The outcomes were compared with the twenty-four compounds which consists of apigenin (>98.0% HPLC), (−)-epigallocatechin gallate (>98.0% HPLC), 3,4-dihydroxybenzoic acid (≥97% T), 4-hydroxybenzoic acid (>99.0% GC, T), hesperidin (>90.0% HPLC, T), chlorogenic acid (>98.0% HPLC, T), caffeic acid (>98.0% HPLC, T), *p*-coumaric acid (>98.0% GC, T), luteolin (>98.0% HPLC), kaempferol (>97.0% HPLC), myricetin (>97.0% HPLC), syringic acid (>97.0% T), ferulic acid (>98.0% GC, T), cinnamic acid (>98.0% HPLC), naringenin (>93.0% HPLC, T), quercetin (>98.0% HPLC, E) and sinapic acid (>99.0% GC, T), genistein (>98.0% HPLC)from Tokyo Chemical Industry (Tokyo, Japan); rutin (≥94% HPLC), gallic acid (97.5–102.5% T), vanillic acid (≥97% HPLC), rosmarinic acid (≥98% HPLC) from Sigma-Aldrich (St. Louis, MO, USA); galangin (≥98.0% HPLC) from Wuhan ChemFaces Bio-chemical Co., Ltd. (Wuhan, China); isorhamnetin (≥99.0% HPLC) from Extrasynthese (Genay, France). All measurements were carried out in triplicate. The results were calculated and reported on mg of compound per 100 g dry weight (mg/100 g DW) [54]. The validation parameters of twenty-four authentic standards of phenolics were shown in the Supplementary Table S1.

### 4.3. Determination of Enzyme Inhibitory Activities

AChE and BChE inhibition were determined using well-established protocols without modifications [55]. For BACE-1 inhibition, we complied with the manufacturer’s instructions (BACE-1 FRET assay kit, Sigma-Aldrich, St. Louis, MO, USA). In brief, for the AChE assay, 40 µL of either DE extract, donepezil, quercetin, and kaempferol were mixed with 50 µL of 6 mM 5,5′-dithiobis (2-nitrobenzoic acid) (DTNB, an indicator dye), 50 µL of 0.32 mM acetylthiocholine (substrate), and 100 µL of 0.25 µg/mL acetycholinesterase (AChE). For the BChE assay, 40 µL of either DE extract, donepezil, quercetin, and kaempferol were mixed with 50 µL of 6 mM DTNB, 50 µL of 0.4 mM butyrylthiocholine (substrate), and 100 µL of 1.5 µg/mL butyrylcholinesterase (BChE). Both enzymatic reactions were recorded at 412 nm using a SynergyTM HT 96-well UV-visible spectrophotometer (BioTek Instruments, Inc., Winooski, VT, USA). The enzyme inhibitory percentage was calculated as follows:

(1) $$\%\;\mathrm{inhibition}=\left(1-\;\frac{B-b}{A-a}\right)\times 100,$$

where *A* is the initial velocity of the reaction with enzyme, *a* is the initial velocity of the reaction without enzyme, *B* is the initial velocity of the enzyme reaction with extract, and *b* is the initial velocity of the reaction with extract but without enzyme.

The half-maximal inhibitory concentration (IC_50_) against AChE, BChE and BACE-1 were calculated using GraphPad Prism (version 9.0, La Jolla, CA, USA) as follows:

(2) $$Y=\mathrm{Bottom}+\left(\frac{\mathrm{Top}-\mathrm{Bottom}}{1+10^{((\log{\mathrm{IC}}_{50}-X)\;\times \;(\mathrm{HillSlope}))}}\right),$$

where Y is response, decreasing as X increases, X is log of dose or concentration, Top and Bottom are plateaus in same units as Y, and Hillslope is data slope factor or hill slope, unitless.

### 4.4. Determination Synergistic Effect between Donepezil and DE Extract

The combination index (CI) was calculated using the following equation [56].

(3) $$\mathrm{CI}=\frac{C_{\mathrm{AX}}}{{\mathrm{IC}}_{A}}+\frac{C_{\mathrm{BX}}}{{\mathrm{IC}}_{B}},$$

where C_AX_ and C_BX_ are concentrations of donepezil and DE extract or quercetin or kaempferol used in combination to reach IC_50_. IC_A_ and IC_B_ comprise the IC_50_ values for single donepezil and DE extract or quercetin or kaempferol agents. The description of CI values is shown in Table 6 with minor modification [57,58].

### 4.5. Fly Strains, Culture, and Treatment

Flies, including UAS-APP-BACE-1 (BDSC 33798) were obtained from Bloomington *Drosophila* stock center and GMR-GAL4 was gifted from Dr. Hideki Yoshida, Kyoto Institute of Technology, Japan. All fly stocks were maintained in a standard medium at 25 °C, 60% humidity, and a 12 h/12 h light–dark cycle in constant climate chambers with ICH-compliant light source (BINDER GmbH, Tuttlingen, Germany). Mating between them resulted in F1 progenies expressing human amyloid precursor protein (APP) and β-secretase-1 (BACE-1) in the fly eyes. F1 flies (one-day-old) were treated with several combination of donepezil (2.5 to 10 µM) and DE extract (62.5 to 250 µg/mL). Flies were cultivated for 21 days at 28 °C, with food changes every three days.

### 4.6. Analysis of Drosophila Eyes Using Scanning Electron Microscopy (SEM)

After 21 days of treatment, only male files were anesthetized and coated using a vacuum sputter coater (DV 502) (Denton Vacuum, Moorestown, NJ, USA), at a pressure of 100 psi and a current of 10 mA for 60 s. The external compound eyes were then inspected using JSM-IT510 InTouchScope™ SEM Series (JEOL, Peabody, MA, USA) in a vacuum mode.

### 4.7. Bacterial Reverse Mutation Test (Ames Test)

The DE extract was tested for its mutagenicity following the OECD guideline for testing of chemicals No. 471 [23]. In brief, *Salmonella typhimurium* (TA98, TA100, TA102, TA1535, and TA1537) was cultured in a nutrient broth (12 mL) at 37 °C. Then, 100 µL of the culture (optical density of 0.3–0.4) was added to the tube containing 50 µL of different doses of DE extract and 500 µL of PBS or 500 µL of S9 mixture (Sigma-Aldrich, St. Louis, MO, USA) and then pre-incubated at 37 °C for 20 min. Later, the mixture was added to 2 mL of top agar containing 0.5 mM L-histidine and 0.5 mM D-biotin. The mixture was agitated and poured onto the minimal agar plate and incubated at 37 °C for 48 h. All experiments were conducted in triplicate. The number of revertant colonies per plate was counted. The MR was calculated from the average of the revertant number divided by the average of negative control revertant number [59,60].

## 5. Conclusions

Our results showed that *Diplazium esculentum* (Retz.) Sw. (DE) extract combined with donepezil showed slightly antagonistic properties with AChE, while DE extract and quercetin exhibited synergistic and additive effects with donepezil against BChE and BACE-1, respectively. AD is a disease that originates from various mechanisms; therefore, therapeutic interventions that inhibit these mechanisms demonstrate considerable promise. This study is the first report that shows that DE extract and donepezil inhibit BChE and BACE-1 synergistically and/or additively, indicating a prospective role for DE extract in the treatment of AD when combined with donepezil through multi-targeted therapies (cholinergic hypothesis via BChE and amyloid peptide pathway via BACE-1). However, further investigations in animal models, such as AD mice, are required to support these findings.

## Figures and Tables

**Table 1:** Phenolic profiles of ethanolic extract of *Diplazium esculentum* (DE extract) determined using liquid chromatography–electrospray ionization tandem mass spectrometry (LC–ESI–MS/MS).

Sample	Phenolic Profiles (mg/100 g Dry Weight (DW))	
DE extract	Rutin	6.76 ± 0.25 ^A^
	Galangin	19.20 ± 0.03 ^B^
	Rosmarinic acid	53.88 ± 0.50 ^C^
	Quercetin	87.96 ± 0.09 ^D^
	Kaempferol	167.67 ± 4.77 ^E^

Experimental data are shown as mean ± standard deviation (SD) of triplicate investigations (*n* = 3). Different uppercase letters (A, B, C, D and E) denote significantly different contents of phenolics at *p* < 0.05 using one-way analysis of variance (ANOVA), followed by Duncan’s multiple comparison test.

**Table 2:** Half-maximal inhibitory concentration (IC_50_) of ethanolic extract of *Diplazium esculentum* (DE extract) and its abundantly found phenolics (kaempferol and quercetin) on acetylcholinesterase (AChE), butyrylcholinesterase (BchE), and β-secretase (BACE-1) inhibitory assays compared to an Alzheimer’s disease drug (donepezil).

Sample	Half-Maximal Inhibitory Concentration (IC_50_) (µg/mL)		
	AChE	BChE	BACE-1
DE extract	3026.00 ± 70.22 ^D^	3064.21 ± 83.12 ^D^	384.11 ± 33.12 ^D^
Kaempferol	92.16 ± 2.44 ^B^	52.99 ± 4.84 ^B^	85.70 ± 3.67 ^B^
Quercetin	224.30 ± 49.18 ^C^	165.50 ± 35.57 ^C^	133.43 ± 25.98 ^C^
Donepezil	1.30 ± 0.13 ^A^	1.05 ± 0.14 ^A^	0.55 ± 0.02 ^A^

Experimental data are shown as mean ± standard deviation (SD) of triplicate investigations (*n* = 3). Different uppercase letters (A, B, C, and D) denote significantly different enzyme inhibitions in each column at *p* < 0.05 using one-way analysis of variance (ANOVA), followed by Duncan’s multiple comparison test.

**Table 3:** Qualitative analysis of the interactions between donepezil and ethanolic extract of *Diplazium esculentum* (DE extract), kaempferol, and quercetin on acetylcholinesterase (AChE), butyrylcholinesterase (BChE), and β-secretase (BACE-1) inhibitions.

Sample Combination				Combination Index (CI)	Description
Donepezil (µg/mL)	DE Extract (µg/mL)	Kaempferol (µg/mL)	Quercetin (µg/mL)		
**Acetylcholinesterase inhibition**					
IC_30_ (0.66)	IC_30_ (2350.00)			1.28	Slight antagonism
IC_40_ (0.75)	IC_20_ (1800.00)			1.17	Slight antagonism
IC_40_ (0.75)	IC_30_ (2350.00)			1.35	Slight antagonism
IC_50_ (1.30)	IC_20_ (1800.00)			1.59	Antagonism
IC_40_ (0.75)		IC_20_ (40.50)		1.01	Additive
IC_40_ (0.75)		IC_30_ (57.50)		1.20	Slight antagonism
IC_50_ (1.30)			IC_20_ (100.00)	1.45	Slight antagonism
IC_50_ (1.30)			IC_30_ (200.40)	1.89	Antagonism
**Butyrylcholinesterase inhibition**					
IC_20_ (0.25)	IC_20_ (1600.00)			0.76	Moderate synergism
IC_30_ (0.42)		IC_20_ (24.70)		0.87	Slight synergism
IC_30_ (0.42)		IC_30_ (25.50)		0.88	Slight synergism
IC_20_ (0.25)			IC_20_ (47.20)	0.52	Synergism
**BACE-1 inhibition**					
IC_40_ (0.40)	IC_20_ (70.00)			0.91	Additive
IC_40_ (0.40)	IC_30_ (100.00)			0.99	Additive
IC_40_ (0.40)		IC_20_ (50.00)		1.31	Slight antagonism
IC_40_ (0.40)			IC_20_ (25.00)	0.91	Additive
IC_40_ (0.40)			IC_30_ (35.00)	0.99	Additive

Synergism: CI between 0.3–0.7; moderate synergism: CI between 0.7–0.85; slight synergism: CI between 0.85–0.9; slight antagonism: CI between 1.20–1.45; antagonism: CI between 1.45–3.33, and additive: CI between 0.9–1.10.

**Table 4:** Mutagenicity effects of ethanolic extract of *Diplazium esculentum* (DE extract) on five *Salmonella typhimurium* strains without S9 extract (−S9).

Doses (µg/plate)	TA98		TA100		TA102		TA1535		TA1537	
	Revertant Colonies	MR	Revertant Colonies	MR	Revertant Colonies	MR	Revertant Colonies	MR	Revertant Colonies	MR
Neg	81.83 ± 2.73	1.00 (−)	66.83 ± 5.01	1.00 (−)	369.17 ± 9.79	1.00 (−)	11.33 ± 1.89	1.00 (−)	9.00 ± 1.91	1.00 (−)
10	85.50 ± 2.43	1.04 (−)	68.83 ± 4.63	1.03 (−)	371.67 ± 9.52	1.01 (−)	10.83 ± 2.61	0.96 (−)	10.50 ± 2.14	1.17 (−)
100	83.00 ± 3.42	1.01 (−)	66.50 ± 6.24	1.00 (−)	374.33 ± 9.57	1.01 (−)	10.50 ± 1.71	0.93 (−)	8.83 ± 1.46	0.98 (−)
500	83.67 ± 3.90	1.02 (−)	67.83 ± 5.84	1.01 (−)	368.17 ± 7.60	1.00 (−)	9.00 ± 1.53	0.79 (−)	10.83 ± 1.57	1.20 (−)
1000	83.33 ± 4.57	1.02 (−)	69.33 ± 4.82	1.04 (−)	354.50 ± 9.25	0.96 (−)	10.33 ± 0.94	0.91 (−)	8.67 ± 1.25	0.96 (−)
2000	83.83 ± 2.91	1.02 (−)	67.17 ± 5.96	1.00 (−)	369.00 ± 8.29	1.00 (−)	11.00 ± 1.63	0.97 (−)	9.17 ± 1.77	1.02 (−)
4-NQO	878.67 ± 35.25	10.74 (+)								
NaN_3_			1145.33 ± 52.03	17.14 (+)			214.17 ± 9.51	18.90 (+)		
MMC					955.33 ± 27.85	2.59 (+)				
9-AA									780.00 ± 24.11	86.67 (+)

All data are shown as mean ± standard deviation (SD) of triplicate experiments (*n* = 3). Negative control (Neg) is distilled water used as a solvent control. MR: mutagenicity ratio; positive control: 4-NQO: 4-nitroquinoline-1-oxide; NaN_3_: sodium azide; MMC: mitomycin C; 9-AA: 9-aminoacridine; (−): indicates the mutagenicity ratio (MR) is ≤1; (+): indicates the mutagenicity ratio (MR) is ≥2.

**Table 5:** Mutagenicity effects of ethanolic extract of *Diplazium esculentum* (DE extract) on five *Salmonella typhimurium* strains with S9 extract (+S9).

Doses (µg/plate)	TA98		TA100		TA102		TA1535		TA1537	
	Revertant Colonies	MR	Revertant Colonies	MR	Revertant Colonies	MR	Revertant Colonies	MR	Revertant Colonies	MR
Neg	98.67 ± 5.96	1.00 (−)	87.33 ± 4.38	1.00 (−)	334.00 ± 10.98	1.00 (−)	10.50 ± 1.26	1.00 (−)	9.83 ± 1.57	1.00 (−)
10	99.67 ± 4.68	1.01 (−)	85.33 ± 5.56	0.98 (−)	340.00 ± 12.19	1.02 (−)	11.50 ± 1.86	1.10 (−)	9.00 ± 0.58	0.92 (−)
100	96.67 ± 3.54	0.98 (−)	84.50 ± 4.07	0.97 (−)	346.50 ± 10.89	1.04 (−)	11.17 ± 1.07	1.06 (−)	9.00 ± 1.29	0.92 (−)
500	96.33 ± 4.38	0.98 (−)	83.83 ± 2.67	0.96 (−)	344.83 ± 10.32	1.03 (−)	11.50 ± 1.26	1.10 (−)	9.83 ± 0.90	1.00 (−)
1000	94.33 ± 4.68	0.96 (−)	81.83 ± 3.67	0.94 (−)	345.00 ± 7.37	1.03 (−)	11.33 ± 1.25	1.08 (−)	11.17 ± 1.34	1.14 (−)
2000	96.83 ± 5.43	0.98 (−)	83.83 ± 2.61	0.96 (−)	345.17 ± 8.29	2.88 (−)	10.00 ± 1.83	0.95 (−)	9.83 ± 1.95	1.00 (−)
2-AA	1396.00 ± 48.94	14.15 (+)	876.67 ± 34.21	10.04 (+)	960.67 ± 29.07	3.03 (+)	349.83 ± 18.89	33.32 (+)	203.67 ± 5.56	20.71 (+)

All data are shown as mean ± standard deviation (SD) of triplicate experiments (*n* = 3). Negative control (Neg) is distilled water used as a solvent control. MR: mutagenicity ratio; 2-AA: 2-aminoanthracen; (−): indicates the mutagenicity ratio (MR) is ≤1; (+): indicates the mutagenicity ratio (MR) is ≥2.

**Table 6:** Description of CI index.

CI Values	Description	CI Values	Description
<0.1	Very strong synergism	0.9–1.10	Additive
0.1–0.3	Strong synergism	1.20–1.45	Slight antagonism
0.3–0.7	Synergism	1.45–3.33	Antagonism
0.7–0.85	Moderate synergism	3.3–10	Strong antagonism
0.85–0.9	Slight synergism	>10	Very strong antagonism

## Data Availability

Data are contained within this article and Supplementary Materials.

## Supplementary Materials

The following supporting information can be downloaded at: https://www.mdpi.com/article/10.3390/ph17030341/s1, Supplementary Table S1: The validation parameters of twenty-four authentic standards of phenolics using liquid chromatography-electrospray ionization tandem mass spectrometry (LC-ESI-MS/MS) in selective reaction monitoring (SRM) mode; Supplementary Figure S1: A full liquid chromatography-electrospray ionization tandem mass spectrometry (LC–ESI-MS/MS) chromatograms of ethanolic extract of *Diplazium esculentum* (DE extract) presented five detected phenolic compounds including 1: rutin, 2: rosmarinic acid, 3: quercetin, 4: kaempferol, and 5: galangin.
