# Supplementary material for: Enhancing Therapeutic Efficacy of Donepezil, an Alzheimer’s Disease Drug, by Diplazium esculentum (Retz.) Sw. and Its Phytochemicals
Source: Pharmaceuticals (Basel). 2024 Mar 6;17(3):341. doi: 10.3390/ph17030341 (PMC10975765; doi:10.3390/ph17030341)

pharmaceuticals

Supplementary materials

Enhancing Therapeutic Efficacy of Donepezil, an Alzheimer's Disease Drug, by *Diplazium esculentum* (Retz.) Sw. and Its Phytochemicals

Woorawee Inthachat [1], Boonrat Chantong [2], Pornsiri Pitchakarn [3], Chawalit Takoon [4], Jirarat Karinchai [3], Uthaiwan Suttisansanee [1] and Piya Temviriyanukul [1,*]

[1] Food and Nutrition Academic and Research Cluster, Institute of Nutrition, Mahidol University, Salaya, Phuttamonthon, Nakhon Pathom 73170, Thailand.; woorawee.int@mahidol.ac.th (WI); uthaiwan.sut@mahidol.ac.th (U.S.);
[2] Department of Pre-clinical and Applied Animal Science, Faculty of Veterinary Science, Mahidol University, Salaya, Phutthamonthon, Nakhon Pathom 73170, Thailand.; boonrat.cha@mahidol.ac.th (B.C.)
[3] Department of Biochemistry, Faculty of Medicine, Chiang Mai University, Chiang Mai 50200, Thailand.; pornsiri.p@cmu.ac.th (P.P.); jirarat.ka@cmu.ac.th (J.K.)
[4] Mahidol University Frontier Research Facility (MU-FRF), Mahidol University, Nakhon Pathom 73170, Thailand; chawalit.tak@mahidol.ac.th (C.T.)
* Correspondence: piya.tem@mahidol.ac.th (P.T.)

Supplementary Table S1:

The validation parameters of twenty-four authentic standards of phenolics using liquid chromatography-electrospray ionization-tandem mass spectrometry (LC-ESI-MS/MS) in selective reaction monitoring (SRM) mode. The validation data were obtained from our previus work (Sirichai et.al., 2022).

Compounds	Retention time (min)	Standards	Linear range (µg/mL)	Linear regression equation	Correlation coefficient (R^2)	LOD (µg/mL)	LOQ (µg/mL)	%RSD (Inter-day)	%Recovery		
									Low level (µg/mL)	Medium level (µg/mL)	High level (µg/mL)
1	0.44	Epigallocatechin gallate	0.125–40	y =8533x + 1053.4	0.9985	0.067	0.230	0.023	91.84	85.36	91.37
2	0.564	Gallic acid	0.195–25	y =3323.1x – 2100.4	0.9984	0.04	0.14	0.01	113.05	118.57	109.12
3	0.803	3,4-Dihydroxybenzoic acid	0.195–25	y =11490x – 10877	0.9935	0.010	0.034	0.003	90.59	85.75	89.75
4	0.922	Chlorogenic acid	0.3125–40	y =8377.5x – 3623.5	0.9934	0.017	0.055	0.006	91.94	87.50	95.02
5	1.16	4-Hydroxybenic acid	0.3125–40	y =2482.6x– 3998.4	0.9917	0.027	0.090	0.009	109.67	103.60	101.28
6	1.40	Caffeic acid	0.3125–40	y =12328x–19725	0.9918	0.010	0.035	0.003	105.36	93.98	87.41
7	1.539	Syringic acid	3.125–100	y =68.091x + 230.43	0.9955	0.582	1.939	0.194	116.35	97.42	94.91
8	1.63	Vanillic acid	2.5–100	y =213.67x – 975.72	0.9900	0.15	0.48	0.05	99.86	101.76	100.12
9	2.452	*p*-Coumaric acid	0.3125–40	y =8532.4x–13559	0.9910	0.013	0.042	0.004	88.22	81.36	98.05
10	2.737	Rutin	0.009–1.25	y =49729x – 33.064	0.9999	0.001	0.005	0.0005	94.63	114.00	108.73
11	2.772	Sinapic acid	0.39–25	y =1592.6x – 832.22	0.9977	0.026	0.086	0.009	81.34	92.16	84.22
12	2.851	Ferulic acid	1.56–100	y =559.03x – 1819.2	0.9947	0.155	0.518	0.052	91.51	89.24	93.10
13	3.41	Hesperidin	0.25–40	y =838.63x – 242.2	0.9986	0.07	0.22	0.02	100.43	104.06	108.60
14	3.431	Myricetin	1.25–40	y =303.47x – 601.81	0.9976	0.261	0.871	0.087	113.07	81.77	91.12
15	3.528	Rosmarinic acid	0.3125–40	y =4322.4x-3744.1	0.9956	0.07	0.25	0.02	92.45	106.35	99.62
16	4.158	Luteolin	0.195–12.5	y =8381.9x – 5000.7	0.9945	0.015	0.050	0.0005	84.21	96.21	107.09
17	4.185	Quercetin	0.05–12.5	y =2934x + 917.17	0.9937	0.05	0.18	0.02	83.36	115.06	95.74

Supplementary Table S1 (Cont.):

The validation parameters of twenty-four authentic standards of phenolics using liquid chromatography-electrospray ionization-tandem mass spectrometry (LC-ESI-MS/MS) in selective reaction monitoring (SRM) mode. The validation data were obtained from our previus work (Sirichai et.al., 2022).

Compounds	Retention time (min)	Standards	Linear range (μg/mL)	Linear regression equation	Correlation coefficient (R^2)	LOD (μg/mL)	LOQ (μg/mL)	%RSD (Inter-day)	%Recovery		
									Low level (μg/mL)	Medium level (μg/mL)	High level (μg/mL)
18	4.522	Cinnamic acid	0.039–10	y =6631.9x – 866.59	0.9964	0.049	0.163	0.016	101.94	98.84	95.85
19	4.689	Apigenin	0.34–11	y =1790.7x – 287.7	0.9997	0.127	0.424	0.042	88.84	106.89	114.79
20	4.693	Genistein	0.625–40	y =1247.2x – 1747.1	0.9977	0.049	0.163	0.016	95.33	101.49	11633
21	4.705	Naringenin	0.0008–5	y =16755x + 443.03	0.9932	0.003	0.011	0.001	117.92	96.26	111.08
22	4.79	Kaempferol	0.25–10	y =1006.8x – 346.28	0.9905	0.122	0.406	0.041	92.35	107.69	102.17
23	4.878	Isorhamnetin	0.0098–2.5	y =12698x + 586.16	0.9945	0.016	0.052	0.005	113.57	105.88	111.14
24	6.146	Galangin	0.3125–40	y =5012.1x – 9354.7	0.9879	0.010	0.035	0.003	84.01	112.92	115.80

Reference: Sirichai, P.; Kittibunchakul, S.; Thangsiri, S.; On-Nom, N.; Chupeerach, C.; Temviriyanukul, P.; Inthachat, W.; Nuchuchua, O.; Aursalung, A.; Sahasakul, Y.; et al. Impact of Drying Processes on Phenolics and In Vitro Health-Related Activities of Indigenous Plants in Thailand. Plants 2022, 11, 294. https://doi.org/10.3390/plants11030294.

pharmaceuticals

MDPI

Supplementary Figure S1:

A full liquid chromatography-electrospray ionization tandem mass spectrometry (LC–ESI-MS/MS) chromatograms of ethanolic extract of *Diplazium esculentum* (DE extract) presented five detected phenolic compounds including 1: rutin, 2: rosmarinic acid, 3: quercetin, 4: kaempferol, and 5: galangin.

The outcomes were compared with the 24 compounds which consists of apigenin (>98.0% HPLC), (-)-epigallocatechin gallate (>98.0% HPLC), 3,4-dihydroxybenzoic acid (≥97% T), 4-hydroxybenzoic acid (>99.0% GC, T), hesperidin (>90.0% HPLC, T), chlorogenic acid (>98.0% HPLC, T), caffeic acid (>98.0% HPLC, T), *p*-coumaric acid (>98.0% GC, T), luteolin (>98.0% HPLC), kaempferol (>97.0% HPLC), myricetin (>97.0% HPLC), syringic acid (>97.0% T), ferulic acid (>98.0% GC, T), cinnamic acid (>98.0% HPLC), naringenin (>93.0% HPLC, T), quercetin (>98.0% HPLC, E) and sinapic acid (>99.0% GC, T), genistein (>98.0% HPLC)from Tokyo Chemical Industry (Tokyo, Japan); rutin (≥94% HPLC), gallic acid (97.5–102.5% T), vanillic acid (≥97% HPLC), rosmarinic acid (≥98% HPLC) from Sigma-Aldrich (St. Louis, MO, USA); galangin (≥98.0% HPLC) from Wuhan ChemFaces Bio-chemical Co., Ltd. (Hubei, China); isorhamnetin (≥99.0% HPLC) from Extrasynthese (Genay, France).

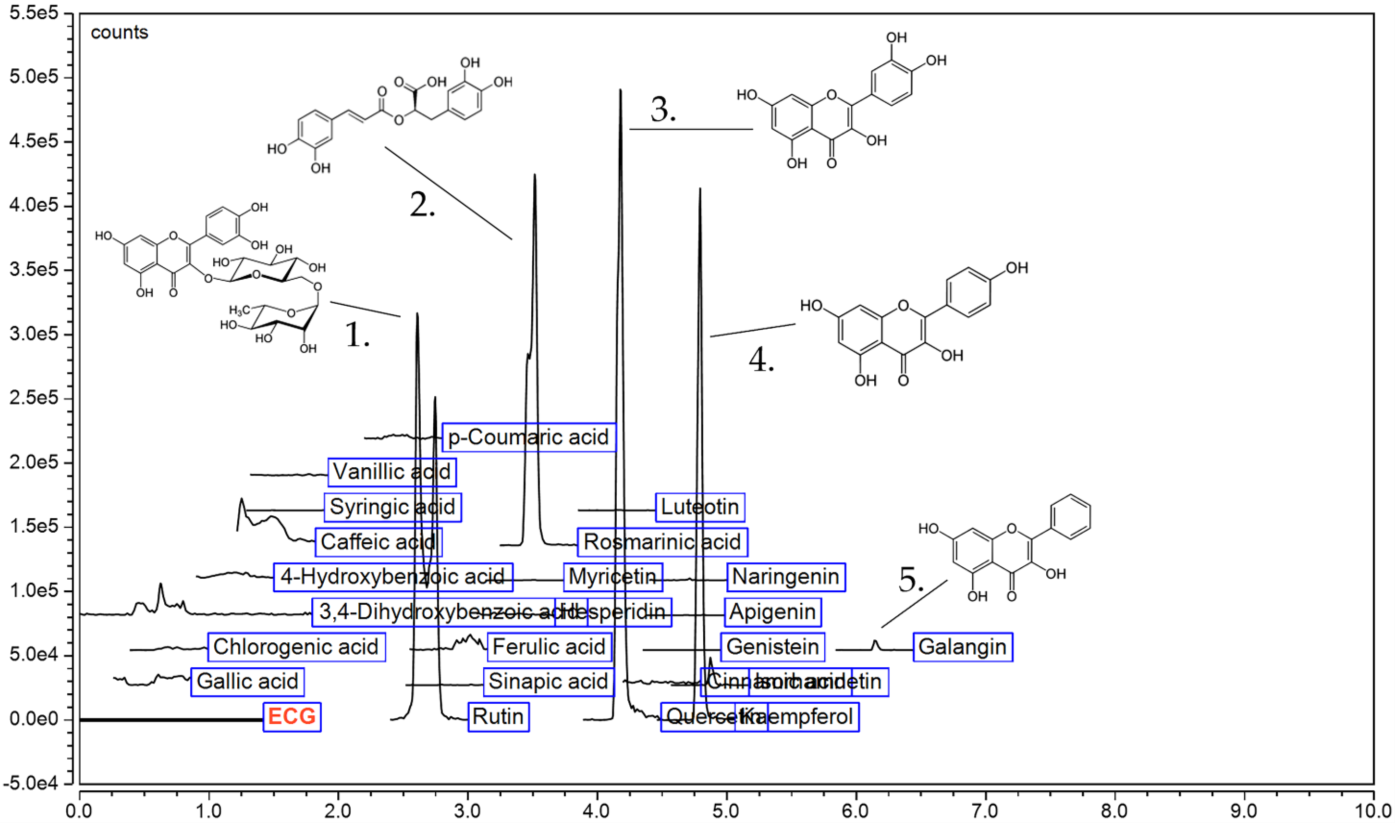

Supplement: Supplementary file 1 [file pharmaceuticals-17-00341-s001.zip › pharmaceuticals-2783660-supplementary.pdf]
